# A Pilot Investigation of the Association Between Vpr Amino Acid Substitutions and Peripheral Immune Marker Levels in People With Human Immunodeficiency Virus: Implications for Neurocognitive Impairment

**DOI:** 10.1093/ofid/ofae111

**Published:** 2024-02-27

**Authors:** Levanco K Asia, Esmé Jansen Van Vuren, Iolanthé M Kruger, Monray E Williams

**Affiliations:** Human Metabolomics, North-West University, Potchefstroom, South Africa; Hypertension in Africa Research Team, North-West University, Potchefstroom, South Africa; South African Medical Research Council, Unit for Hypertension and Cardiovascular Disease, North-West University, Potchefstroom, South Africa; Africa Unit for Transdisciplinary Health Research, North-West University, Potchefstroom, South Africa; Human Metabolomics, North-West University, Potchefstroom, South Africa

**Keywords:** HIV neuropathogenesis, inflammation, polymorphisms, sequence variations, Vpr

## Abstract

**Background:**

Subtype-specific amino acid variations in viral proteins of human immunodeficiency virus type 1 (HIV-1) influence disease progression. Furthermore, Vpr sequence variation correlates with chronic inflammation, a central mechanism in HIV-1 (neuro)pathogenesis. Nevertheless, no clinical study has investigated the link between Vpr sequence variation and peripheral inflammation in people with HIV (PWH). The aim of this pilot study was to ascertain whether specific Vpr amino acid variants were associated with immune markers in PWH.

**Methods:**

We included a unique cohort of 48 treatment-naive South African PWH to determine the association between blood-derived Vpr sequence variation and peripheral immune marker levels using Sanger sequencing and enzyme-linked immunosorbent assay analysis, respectively.

**Results:**

Our findings indicate that among the many neuropathogenic Vpr amino acid variants and immune markers examined, after applying Bonferroni corrections (*P* = .05/3) and adjusting for sex and locality, soluble urokinase plasminogen activator receptor (suPAR) was nearing significance for higher levels in participants with the G41 amino acid variant compared to those with the S41 variant (*P* = .035). Furthermore, amino acid variations at position 41 (between G41 and S41) exhibited a significant association with suPAR (adjusted *R*^2^ = 0.089, β = .386 [95% confidence interval, .125–3.251]; *P* = .035).

**Conclusions:**

These findings suggest that Vpr amino acid sequence variations might contribute to dysregulated inflammation, which could explain the observed association between specific Vpr variants and HIV-1 (neuro)pathogenesis found in prior research. These Vpr variants merit further investigation to fully understand their roles in HIV-1 pathogenesis and neuropathogenesis.

## Teaser

In South African people with HIV, the presence of Vpr amino acid G41 showed significant associations with higher levels of soluble urokinase plasminogen activator receptor compared to other amino acids at these positions.

Human immunodeficiency virus (HIV) remains of global concern, with approximately 37.7 million people living with HIV (PWH), of which 1.5 million were newly infected in 2021 [1]. Dysregulated inflammation is a common hallmark of HIV pathogenesis and an accelerator of non-AIDS-related events and is one of the driving forces of CD4^+^ T-cell depletion [2, 3]. Similarly, dysregulated peripheral and central nervous system (CNS) inflammation has been associated with HIV type 1 (HIV-1) neuropathogenesis [4]. HIV can invade the CNS when HIV-infected monocytes cross the blood-brain barrier (BBB), acting as a “Trojan horse” [5]. After penetrating the BBB, these monocytes transform into macrophages, initiating an immune response in the CNS ultimately leading to neuronal damage and manifesting clinically as HIV-associated neurocognitive disorder (HAND) [5, 6]. Despite advancements in treating HIV infections and HAND with combination antiretroviral therapy (cART), chronic inflammation persists, contributing to the ongoing presence of comorbid conditions, including mild forms of HAND [6].

HIV exhibits high genetic variability, influencing the mechanisms related to both HIV-1 pathogenesis and neuropathogenesis [7, 8]. HIV is categorized into two main types, HIV-1 and HIV-2, due to its high genetic variability. HIV-1 is more prevalent globally [7]. HIV-1 further consists of groups M, N, O, and P with group M being the most abundant globally [7]. Based on differences in the viral genome, group M can be further subdivided into subtypes A, B, C, D, F, G, H, J, and K [7]. Geographically, these subtypes are linked to specific regions worldwide [7]. Subtype B is predominantly found in the United States of America and Europe, whereas subtype C is prevalent in southern Africa [7]. Subtype C accounts for the majority of HIV-1 cases, representing approximately 40%–60% of global infections [7]. Notably, it is the dominant subtype in South Africa [7]. Furthermore, the prevalence of HIV and HAND varies across these different HIV-1 subtypes [7, 8].

The differences among subtypes also manifest in changes in the amino acid sequences of HIV-1 viral proteins, with different subtypes exhibiting sequence variations in the crucial HIV viral proteins Tat, Vpr, Pol, Nef, and gp120, resulting in distinct functionalities of the viral proteins across the subtypes [9–11]. In particular, amino acid sequence variation in several HIV-1 viral proteins was previously associated with a differential HIV-1 pathogenesis, inflammation, and the development of HAND [8, 11–13].

Despite the numerous mechanisms contributing to HIV pathogenesis and neuropathogenesis, it is proposed that in the modern cART era, chronic dysregulated inflammation remains a principal factor driving the continual emergence of milder HAND forms [14]. It is well-established that inflammation within the CNS and brain tissue is linked to HIV-associated encephalopathy and the clinical presentation of neurocognitive impairment in PWH [15]. Given that the cerebrospinal fluid (CSF) is in direct contact with the CNS, it can reflect the biochemical milieu of the CNS during disease states [16]. However, the invasiveness of CSF collection means it is not routinely collected for research purposes. This limitation makes peripheral blood a compelling alternative for exploring potential CNS pathologies. In line with this, evidence suggests that peripheral inflammation can shed light on the neuroinflammatory and/or neuropathological profile. A study by Bryant and colleagues reported that plasma-soluble CD163 is associated with postmortem brain pathology in HIV-1 infection [17]. A study by Gisslén and colleagues indicated that concentrations of the neurofilament light chain protein in plasma and CSF—a sensitive marker of neuronal injury—were highly correlated (*r* = 0.89, *P* < .0001) [18]. This suggests that peripheral inflammation may (1) reflect CNS activity to some extent, (2) correspond with inflammation levels in the CNS, and (3) serve as an alternative approach to investigating CNS pathological states related to inflammation. Notably, the activity of the HIV-1 viral proteins is recognized as significant inducers of the dysregulated inflammatory profiles observed in PWH.

However, among all the HIV-1 viral proteins investigated [19, 20], fewer studies have investigated Vpr despite its function in clinical outcomes in PWH [9, 21, 22]. Vpr presence and sequence variation are associated with various clinical outcomes related to HIV-1 pathogenesis, disease progression, treatment efficacy, and neurological outcomes [9–13]. Supporting this notion, fundamental studies have found that particular amino acids within Vpr can induce higher levels of inflammatory markers [22]. However, no clinical studies to date have demonstrated a link between Vpr sequence variation and immune markers. Thus, this study aimed to investigate how Vpr sequence variations, particularly in amino acids previously linked with HIV neuropathogenesis (at positions 22, 41, 45, and 55), associate with inflammation. The insights from this research may shed light on the observed correlations between Vpr amino acid sequence variations and inflammation in PWH.

## MATERIALS AND METHODS

### Study Participants

The Prospective Urban and Rural Epidemiology (PURE) study is a multinational prospective study designed to examine the development of cardiovascular disease (CVD) in individuals from 27 high-, middle-, and low-income countries [23]. In particular, the PURE study investigated potential risk factors of CVD, which included HIV-1 infection and peripheral inflammation [24]. As part of this larger study, individuals of African descent, both men and women aged >30 years, were recruited from 2 urban and 2 rural communities in the North-West Province of South Africa. Baseline data collection, encompassing 2010 participants, was voluntarily conducted in 2005. Subsequent follow-up data were gathered in 2010 (n = 1288) and 2015 (n = 923). Participants were rediagnosed in 2010 to confirm their HIV status. Therefore, individuals who screened positive for HIV-1 and were treatment naive at the time of data collection in 2010 were included in this study (n = 103). HIV-1 sequencing was successful for only n = 48 PWH; thus, only these were included in the subsequent analysis. Despite the high prevalence rate of HIV and CVD multimorbidity in South Africa, it was previously shown that the participants involved in this study did not exhibit a worse CVD risk profile than those without HIV [25–27]. This study presents a unique cohort of individuals who have not been treated for HIV, allowing us to investigate the inflammatory/immune profile without the confounding influence of cART. The study protocol was approved by the Health Research Ethics Committee of the North-West University (NWU-HREC) in South Africa (NWU-00106-22-A and NWU-00106-22-A1) and substudy (NWU-00106-22-A1-01).

### HIV Status

Participants received counseling by a trained counselor prior to their HIV status determination. The South African Department of Health protocol was used to determine the HIV status using the First Response rapid HIV card test (Premier Medical Corporation Ltd, Daman, India). The test was confirmed with the SD BIOLINE HIV 1/2 3.0 card test (Standard Diagnostics, Inc, Korea). Upon confirmation of a positive result, participants received postcounseling. They were referred to the nearest clinic or hospital for follow-up and CD4^+^ cell count analysis using the flow cytometric method (EPICS XlTM, Beckman Coulter, Fullerton, California).

### Analysis of Immune Markers in Blood Samples

Fasting blood samples were collected and were prepared through centrifugation at a speed of 2000*g* for 15 minutes at 10°C within 2 hours of collection. The samples were then transferred to microfuge tubes, snap-frozen on dry ice, and stored at −80°C in the laboratory until analysis. For samples collected in rural areas, samples were snap-frozen on dry ice and stored at −18°C for a maximum of 5 days until they could be transported to the laboratory and stored at −80°C. For this study, the immune markers soluble urokinase plasminogen activator receptor (suPAR), interleukin 6 (IL-6), high-sensitivity C-reactive protein (hsCRP), soluble CD163 (sCD163), and neutrophil gelatinase–associated lipocalin (NGAL) were selected for investigation. These markers were selected for analysis based on their potential involvement in Vpr-mediated HIV pathogenesis and neuropathogenesis, as reviewed from the scientific literature [9, 28–31]. Independent of HIV, sCD163 is an immune marker that has been demonstrated to play a role in chronic liver disease and insulin resistance [32, 33]. NGAL has been shown to contribute to kidney function and the duration of end-stage renal failure, CVD, and mortality [34, 35]. suPAR has been shown to contribute to acute kidney injury and modulate monocyte function to promote atherosclerosis [36, 37]. IL-6 is a predictor of long-term cardiovascular mortality [38], atherosclerotic CVD, and CVD mortality [39]. hsCRP has been shown to contribute to acute ischemic stroke [40]. suPAR levels were determined from plasma (ethylenediaminetetraacetic acid) samples with the suPARnostiC enzyme-linked immunosorbent assay (ELISA) kit (ViroGates, Copenhagen, Denmark). hsCRP levels were analyzed by particle-enhanced turbidimetric assay, and IL-6 was determined in plasma using an Elecsys 2010 (Roche, Basel, Switzerland) apparatus with the electrochemiluminescence immunoassay method. sCD163 and NGAL were measured using ELISA (DuoSet ELISA, R&D Systems, Minneapolis, United States of America) according to the manufacturer's instructions. All samples were assayed in duplicate. All tests’ intra- and interassay coefficients of variation were within acceptable ranges of <8% and <10%, respectively.

### Viral Protein Analysis

RNA was extracted from 200 μL of prepared plasma using the Quick-RNA Viral Kit (Zymo Research, Irvine, California, United States of America). Total RNA was reverse transcribed by reverse-transcription polymerase chain reaction (PCR) using the ProtoScript II First Strand cDNA Synthesis Kit (New England Biolabs, Ipswich, Massachusetts, United States of America), and DNA was prepared for PCR analysis. The Tat exon 1/Vpr (HXB2 position 4900–6351) was amplified using the primer pair Vif-1 (5′GGGTTTATTACAGGGACAGCA GAG)/CATH-4R (5′-GTACCCCATAATAGACTGTGACC) PCR, respectively. Amplification conditions were held at 94°C for 2 minutes, followed by 40 cycles of denaturing (94°C for 30 seconds), annealing (60°C for 30 seconds), extension (68°C for 2 minutes), and a final extension step for 10 minutes at 68°C. All PCR products were purified with the Nucleospin Gel and PCR clean-up kit, according to manufacturer instructions (Machery-Nagel GmbH & Co. KG, Düren, Germany). All PCR products were sequenced by BigDye Terminator v.3.1 Cycle Sequencing Ready Reaction Kit (ThermoFisher Scientific, Waltham, Massachusetts, United States of America) and analyzed on the ABI Prism 3130xl automated DNA sequencer (Applied Biosystems, Foster City, California). Sequences were analyzed using the Gene Studio professional sequence analysis software (version 2.2). Nucleotide sequences were translated into amino acid sequences using Expasy translate (https://web.expasy.org/translate/), and the key mutations of Vpr were highlighted. Sequences are available in GenBank under the accession numbers OR621303–OR621349.

### Statistical Analysis

All analyses were conducted using SPSS software version 27 (IBM SPSS, Armonk, New York). *P* values were considered statistically significant for all analyses at <.05. All variables were assessed for normality by the visual inspection of QQ plots using descriptive statistics. Data distribution for age and the immune markers suPAR, hsCRP, IL-6, NGAL, and sCD163 were skewed. Therefore, the data was log-transformed before statistical analyses.

As the primary aim, we examined whether peripheral inflammation levels differ between groups divided by different Vpr sequence variations. We employed composite risk scores for Vpr variants, similar to a previous study [41]. We classified amino acids based on their potential influence on mechanisms related to the development of HAND, assigning higher scores to indicate a greater risk. The Vpr amino acids designated as high risk (score = 2) were isoleucine (I) 22, serine (S) 41 or asparagine (N) 41, tyrosine (Y) 45, and alanine (A) 55, while low risk (score = 0) was assigned to leucine (L) 22, glycine (G) 41, histidine (H) 45, and threonine (T) 55. Any other amino acids at the investigated positions were considered neutral risk (score = 1). These selected amino acids have demonstrated influence on neuropathological mechanisms and neurological outcomes in PWH, thus warranting further analysis. A composite “Vpr risk score” was generated by summing the assigned values for each Vpr amino acid variant. Scores ranging from 0 to 3 were considered low-neutral, while scores from 4 to 8 were regarded as high-neutral. The χ^2^ test was used to assess differences in sex, smoking status, alcohol use, and locality between groups. Independent-sample *t* tests were conducted to determine differences in study characteristics (age and CD4^+^ counts) and immune marker levels between the high-neutral and low-neutral groups. A Pearson correlation analysis was used to determine covariates by determining correlations between sociodemographic variables (age, sex, smoking status, alcohol use, and locality), and immune markers. Analyses of covariance (ANCOVA) were performed to compare the immune marker levels between Vpr high-neutral and low-neutral groups, while adjusting for sex and locality. Pearson correlations were used to determine correlations between Vpr risk score and immune marker levels. Multiple regression analysis was used to determine associations between Vpr risk score and immune marker levels after adjusting for potential covariates (locality and sex).

As a secondary aim, we wanted to evaluate whether specific immune marker levels could be related to single amino acid variants. Therefore, we stratified participants into groups: I22 versus L22, N/S41 versus G41, Y45 versus H45, and A55 versus T55. At position 41, we compared G versus N and G versus S, respectively. Pearson χ^2^ tests were used to test group differences for sex and locality between Vpr amino acid variants. Independent-sample *t* tests determined differences in study characteristics (age and CD4^+^ counts) and immune marker levels between Vpr amino acid variants. A Bonferroni correction was used to account for the number of immune markers tested (α/n = .05/5 = .01) in the secondary aim. An ANCOVA was performed to compare the immune marker levels between Vpr variants, while adjusting for sex and locality. A Bonferroni correction was used to account for the number of tests (α/n = .05/3 = .017) within the model. Multiple regression analysis was used to determine associations between Vpr amino acid variance at specific positions and immune marker levels after adjusting for potential covariates (sex and locality).

## RESULTS

### Study Characteristics

This study encompassed a sample of 48 treatment-naive South African subtype C participants, with an average age of 47.46 (standard deviation [SD], 7.604) years. Men constituted only 25% of the participants, as depicted in Table 1. While the primary study did not record any viral load data, it should be noted that all participants were treatment naive at the time of sample collection. CD4^+^ count data were available for 67% of the participants, showing a mean value of 292.00 (SD, 128.41) cells/μL. Stratifying participants based on Vpr amino acid variant risks revealed that the Vpr high-neutral group consisted of 26 participants, while the Vpr low-neutral group had 22 participants (Table 1). No significant differences in study characteristics (age, sex, CD4^+^ count, and locality) were observed between the Vpr high-neutral and Vpr low-neutral groups (Table 1). A Pearson correlation analysis revealed that among all variables (age, sex, smoking status, alcohol use, and locality), only locality and sex were correlated with specific markers. Consequently, we chose to include only these covariates in the relevant analyses.

**Table 1.** Study Characteristics of People With Human Immunodeficiency Virus

Characteristic	People With HIV (N = 48)		
Age, y, mean (SD)	47.46 (7.604)		
Sex, female, No. (%)	36 (75%)		
CD4^+^ count, cells/μL, mean (SD)	292.00 (128.409)		
Locality, rural, No. (%)	28 (58.3%)		
	Vpr High-Neutral (n = 26)	Vpr Low-Neutral (n = 22)	*P* Value
Age, y, mean (SD)	46.23 (7.016)	48.91 (7.72)	ns
Sex, female, No. (%)	18 (69%)	18 (82%)	ns
CD4^+^ count, cells/μL, mean (SD)	299.8 (145.43)	285.5 (115.5)	ns
Locality, rural, No. (%)	13 (50%)	15 (68%)	ns
IL-6, pg/mL, mean (SD)	8.28 (11.37)	7.84 (6.9)	ns
hsCRP, mg/L, mean (SD)	11.39 (28.18)	12.23 (12.23)	ns
suPAR, ng/mL, mean (SD)	4.26 (1.8)	4.8 (2.01)	ns
NGAL, ng/mL, mean (SD)	51.7 (16.4)	53.8 (26.6)	ns
sCD163, ng/mL, mean (SD)	746. 1 (319.2)	792 (420.2)	ns

Logarithmically transformed variables have been back-transformed for presentation purposes.

Abbreviations: HIV, human immunodeficiency virus; hsCRP, high-sensitivity C-reactive protein; IL-6, interleukin 6; NGAL, neutrophil gelatinase–associated lipocalin; ns, not significant; sCD163, soluble CD163; SD, standard deviation; suPAR, soluble urokinase plasminogen activator receptor.

### Immune Marker Levels in the Vpr High-Neutral Group Versus Vpr Low-Neutral Group

Independent sample *t* tests revealed no significant differences in immune marker levels between the Vpr high-neutral and Vpr low-neutral groups (Table 1). To explore the potential influence of covariates on the reported nonsignificant findings, we performed an ANCOVA analysis. Upon controlling for locality and sex using ANCOVA, the levels of IL-6 (*P* = .911), hsCRP (*P* = .744), suPAR (*P* = .219), NGAL (*P* = .826), and sCD163 (*P* = .481) remained statistically nonsignificant between the Vpr high-neutral group and the Vpr low-neutral group.

### Correlation Between Vpr Risk Score and Immune Marker Levels

We aimed to determine if there was a correlation between Vpr risk score and immune marker levels. Using Pearson correlations, we observed no significant correlations between the Vpr risk score and immune markers, namely hsCRP (*R* = −0.091, *P* = .538), IL-6 (*R* = −0.073, *P* = .621), suPAR (*R* = −0.200, *P* = .173), NGAL (*R* = −0.021, *P* = .888), and sCD163 (*R* = −0.103, *P* = .486). Furthermore, a multiple regression analysis that adjusted for covariates (locality and sex) revealed no significant association between the Vpr risk score and hsCRP (adjusted [adj] *R*^2^ = −0.057, β = −.097; 95% confidence interval [CI], −7.8 to 4.0; *P* = .523), IL-6 (adj *R*^2^ = −0.008, β = −.098; 95% CI, −2.2 to 1.1; *P* = .510), suPAR (adj *R*^2^ = 0.021, β = −.223; 95% CI, −.6 to .08; *P* = .133), NGAL (adj *R*^2^ = −0.047, β = −.100; 95% CI, −.09 to .005; *P* = .510), and sCD163 (adj *R*^2^ = 0.066, β = −.067; 95% CI, −.11 to .07; *P* = .638).

### Immune Marker Levels Between Vpr Amino Acid Variants

As an alternative to creating composite risk Vpr groups as described above, our secondary aim was to examine whether we could link immune marker levels to specific amino acid variants (Table 2). We stratified participants according to Vpr amino acid variants at positions 22 (n = 44), 41 (n = 40), 45 (n = 42), and 55 (n = 43). When stratifying participants according to the Vpr amino acids variants, there were no significant differences between study characteristics (age, sex, CD4^+^ count, and locality) (Table 2). After applying Bonferroni correction of *P* = .05/5, only suPAR levels were found to be significantly higher in the G41 group compared to the N41 group (*P* = .008; Table 2). However, after applying Bonferroni correction of *P* = .05/2 and when adjusting for locality and sex through ANCOVA, suPAR was nearing significance for higher levels in the G41 group compared to the S41 group (*P* = .035). However, suPAR was no longer significantly higher in the G41 group compared to the N41 group (*P* = .115).

**Table 2.** Characteristics of Human Immunodeficiency Virus–Positive Participants and Immune Marker Levels When Participants Were Stratified According to Specific Amino Acid Variants

Characteristic	Position 22 (n = 44)		*P* Value	Position 41 (n = 40)			*P* Value	Position 45 (n = 42)		*P* Value	Position 55 (n = 43)		*P* Value
Amino acid^a^	I	L		G	N	S		Y	H		A	T	
No. (%)	23 (52)	21 (48)		21 (53)	8 (20)	11 (27.5)		25 (60)	17 (40)		11 (26)	32 (74)	
Age, y	48.22 (8.2)	46.9 (7.4)	.581	47.9 (6.8)	46.13 (6.7)	46.3 (8.7)	.544/.574	48.32 (8.4)	46.88 (5.8)	.545	47.45 (6.8)	47.28 (7.7)	.948
Sex (female), No. (%)	16 (70)	17 (81)	.862	15 (71)	7 (87.5)	6 (55)	.296	20 (80)	11 (65)	.268	7 (64)	27 (84)	.145
CD4^+^ count	274.80 (119.6)	314.4 (137.9)	.416	292 (133.8)	233.6 (60.2)	357.75 (155.3)	.366/.380	288.07 (101)	279.17 (143)	.851	258.7 (105.8)	317.9 (128.0)	.307
Locality (rural), No. (%)	10 (43)	14 (67)	.123	13 (62)	5 (63)	7 (64)	.995	13 (52)	11 (65)	.618	7 (64)	17 (53)	.800
suPAR, ng/mL	4.51 (1.7)	4.76 (2.2)	.685	5.3 (2.4)	3.65 (0.7)	3.8 (0.9)	**.008**/**.018**	4.98 (2.4)	3.87 (0.8)	.037	5.41 (2.3)	4.12 (1.5)	.056
IL-6, pg/mL	7.93 (6.5)	9 (12.7)	.717	10.5 (13.4)	5.79 (3.57)	6.1 (4.2)	.346/.305	10.62 (12.5)	5.04 (3.1)	.041	13.08 (16.7)	6.18 (5.4)	.166
hsCRP, mg/L	16.19 (40.0)	10.44 (28.9)	.591	19.38 (48.2)	13.17 (21.5)	3.12 (3.6)	.731/.139	20.01 (45.2)	4.68 (5.1)	.104	18.67 (39.4)	11.3 (34.2)	.556
NGAL, ng/mL	57.8 (20.1)	45.5 (21.8)	.027	54.4 (26.8)	55.3 (17)	48.7 (15.3)	.661/.746	54 (22.6)	50.2 (22.3)	.570	50.9 (27.9)	55 (19)	.333
sCD163, ng/mL	801.4 (366.7)	783 (379.8)	.728	759.8 (376)	654.3 (398.1)	807.4 (249.1)	.305/.465	775.3 (391.7)	631.8 (274.2)	.293	791.3 (292)	747.1 (383.9)	.462

Data are presented as mean (standard deviation) unless otherwise indicated. Logarithmically transformed variables have been back-transformed for presentation purposes. At position 41, we compared G vs N and G vs S, respectively. Statistically significant findings are presented in bold.

Abbreviations: hsCRP, high-sensitivity C-reactive protein; IL-6, interleukin 6; NGAL, neutrophil gelatinase–associated lipocalin; sCD163, soluble CD163; suPAR, soluble urokinase plasminogen activator receptor.

^a^Amino acids: A, alanine; G, glycine; H, histidine; I, isoleucine; L, leucine; N, asparagine; S, serine; T, threonine; Y, tyrosine.

Furthermore, we aimed to evaluate the relationships between the Vpr amino acid variance at specific positions and immune marker levels while adjusting for covariates. To do this, we adjusted for covariates, namely locality and sex, using multiple regression analyses. Across all Vpr amino acid positions investigated, only variance at position 41 (between G41 and S41) exhibited a significant association with suPAR (adj *R*^2^ = 0.089, β = .386; 95% CI, .125–3.251; *P* = .035).

## DISCUSSION

In this study, we aimed to investigate whether variations in the HIV-1 Vpr amino acid sequence, especially at amino acid positions previously associated with neurological outcomes (namely, positions 22, 41, 45, and 55), would influence the levels of peripheral immune markers sCD163, NGAL, hsCRP, suPAR, and IL-6 in PWH. This was in aim of providing commentary on its role on potential underlying HIV-1–related pathology. Among all the Vpr amino acids and immune markers investigated, variation at position 44 associated with suPAR, which was shown to be higher in the G41 group when compared to the S41 group after adjustment for covariates.

The previous study by McMullen and colleagues has shown that the Vpr G41 amino acid was associated with nonischemic stroke in PWH [42], also suggesting that Vpr G41 may be a low-risk Vpr variant. In the same cohort of South African participants, the S41 amino acid was associated with ischemic stroke in PWH [42], suggesting that S41 may be a high-risk Vpr variant. However, another study conducted in the United States found that in PWH, the S41 amino acid is associated with a decreased and less pronounced neurocognitive deficit (lower global deficit scores) [21]. The contradiction between these findings may be due to the difference in geographical regions and, most likely, different HIV-1 subtypes [43]. The study by Dampier and colleagues reported that the N41 amino acid was associated with more pronounced neurocognitive deficits (higher global defecit score) [21]. We initially considered the Vpr variant G41 as low risk. However, our findings suggest that it may contribute to increased suPAR levels since suPAR was also higher in this group—a characteristic expected from the high-risk Vpr variants. The limited number of studies reveals no clear consensus on whether the amino acid variation at position 41, particularly G41, may contribute negatively or positively to clinical outcomes in PWH.

Incorporating the existing body of evidence, we introduce the possibility that Vpr G41 might play a role in adverse clinical outcomes among PWH by disrupting the peripheral immune response, particularly involving suPAR. It may also be that amino acid variation at position 41 of Vpr generally may influence the structure-function relationship of Vpr, as amino acid 41 is within the second α-helix (38–50), which is responsible for key mechanisms including regulation of apoptosis, subcellular transport [44], and virion incorporation of Vpr [45]. It is established that hydrophilic amino acids, such as N41 and S41, are situated on a specific side of the helix [46]. Altering this configuration to a smaller amino acid like G41 could potentially impact the arrangement of the Vpr α-helices, therefore influencing its potential functioning.

None of the other investigated Vpr amino acid signatures or inflammatory markers demonstrated significance after applying the Bonferroni correction. It is reasonable to speculate that the small number of participants in the included study may have led to low statistical power. However, future studies should explore these Vpr amino acid signatures in larger cohorts, as these signatures have been previously linked to adverse clinical outcomes in PWH [9]. Studies of this nature may help illuminate the reasons behind the observed variations in clinical severities when comparing participants with different HIV subtype infections. The amino acid at position 77 is extensively studied in the context of Vpr sequence variation [9]. However, the majority of participants in this study exhibited a Q at position 77 (83%). Due to the limited variation at this position, we were unable to explore its influence on immune markers.

Furthermore, it is known that HIV infection leads to the production of immune markers, often resulting in adverse clinical outcomes for PWH [2]. Our study revealed an association between the variance at position 41 and suPAR, a marker implicated in various diseases and disorders. Specifically, elevated plasma suPAR levels have been linked to worsened neurological performance in an HIV-treated population [30]. Consistent with this, increased suPAR levels in the CSF have been associated with lower working memory in PWH [47]. This immune marker was also shown to associate with acute kidney injury and modulation of monocyte function, promoting atherosclerosis [36, 37]. Additionally, suPAR was found to be associated with organ failure independent of HIV infection [48].

Our study’s findings suggest that Vpr amino acid variants, specifically those at position 41, may be associated with adverse clinical outcomes in PWH through mechanisms related to dysregulated inflammation, particularly suPAR [47, 49]. While many studies that aimed to link HIV Vpr amino acid variants to clinical outcomes have been conducted in preclinical settings [22], fewer have explored HIV amino acid variations in clinical research [21, 42]. Moreover, even fewer studies have delved into the influence of HIV Vpr amino acid variants on inflammation, especially those concerning amino acid variations at positions 22, 41, 45, and 55 [21, 42].

Given that our cohort was not undergoing any treatment at the time of sample collection, we were uniquely positioned to delineate the relationship between Vpr amino acid sequence variation and systemic inflammation more clearly. However, a more in-depth investigation of these specific Vpr variants is imperative to comprehensively understand their contributions to HIV-1 pathogenesis and possibly neuropathogenesis. Furthermore, most studies examining Vpr sequence variation have been conducted in regions dominated by subtype B [9]. Few investigations have delved into Vpr sequence variation in the southern African context. Thus, our findings enrich the understanding of prevalent sequence variations within this setting. Further research is warranted to corroborate the relationship between Vpr amino acid variants and inflammation in PWH. This could provide deeper insights into the potential impacts of such associations on clinical outcomes in PWH.

This study has several limitations that warrant emphasis. First, the sample size was limited, which might have influenced the reported potential associations. Therefore, our findings should be interpreted in light of this, and future research is recommended to validate our results in larger cohorts. Though we view this as a pilot study, it offers insights into (1) the South African sequence variation, an area with limited investigation, and (2) the association of Vpr sequence variation in a treatment-naive cohort, giving a clearer snapshot of systemic inflammatory profile without the potential confounding effects of cART. An initial objective of the study was to assess the combined impact of various amino acid substitutions on peripheral inflammatory levels, utilizing a composite risk score and categorizing participants into high-neutral and low-neutral Vpr risk groups. Although this approach has been employed previously [41], we recognize a limitation in that certain amino acids may exert a more significant influence than others. Summing amino acids together in this manner might attenuate this effect, potentially contributing to the lack of significance observed in the investigation of Vpr risk categories and risk scores. Additionally, this study focused on sequence variation in a single HIV-1 protein. Other pivotal viral proteins, such as Tat and gp120, might have also exhibited specific variations that could have contributed to the observations reported here. Nonetheless, our approach to examining a single HIV-1 protein and its association with clinical outcomes is consistent with methods used in other studies [21, 50]. In this study, we also evaluated peripheral markers to draw insights into their potential CNS functioning. While it could be argued that peripheral markers might not directly reflect CNS activity, previous studies have documented associations between peripheral markers and clinical HAND. Moreover, a direct correlation exists between the levels of specific markers in the periphery and the CNS, suggesting that our findings may be relevant for investigations into CNS activity [18]. Furthermore, examining the concentration of the marker neurofilament (NFL) in blood within this study could have provided insights into the potential neuropathology of PWH [18]. Therefore, in cases where CSF or neuropsychological data is unavailable, future studies conducted in blood should explore NFL as a potential indicator of neuropathology. Even though we have considered locality in our analyses, other psychosocial determinants of health may have played a role in influencing immune marker levels and the observed findings. Previous research has demonstrated that mental health, trauma, and stress can impact inflammation in PWH [51–54] and therefore, our findings should be interpreted in relation to this.

## CONCLUSIONS

Variations in the Vpr amino acid sequence have been previously linked to neurocognitive impairment in PWH. Yet, the mechanism driving this association remains unclear. Given the pivotal role of dysregulated inflammation in the development of HAND, we hypothesized that neuropathogenic Vpr amino acid variants might correlate with specific immune markers, suggesting a potential role in the onset of HAND. In this study, we found that the Vpr variant at position 41 was associated with suPAR and that suPAR was higher in PWH with the G41 variant. Therefore, variations in the Vpr amino acid sequence might be related to dysregulated inflammation and the subsequent disease progression. The Vpr signatures highlighted here warrant further investigation to fully understand their roles in HIV-1 pathogenesis and neuropathogenesis.

## Notes

***Acknowledgments.*** We thank the PURE-SA research team; the field workers and office staff in the Africa Unit for Transdisciplinary Health Research, North-West University, South Africa; and Dr S. Yusuf and the PURE project staff at the Population Health Research Institute (Hamilton Health Sciences and McMaster University, Ontario, Canada).

***Author contributions.*** L. K. A. and M. E. W. contributed to conceptualization, investigation, methodology, formal analysis, data curation, and writing. M. E. W. also handled project administration, funding acquisition, and supervision. E. J. V. V. contributed to formal analysis, writing (both original draft and review and editing), and supervision. I. M. K. provided resources and contributed to writing (review and editing).

***Data availability.*** All data are available in the article.

***Patient consent.*** All participants provided written informed consent, and their information was anonymized to the greatest extent possible.

***Financial support*.** This original work was financially supported by SANPAD (South Africa–Netherlands Research Programme on Alternatives in Development); the South African National Research Foundation (NRF GUN numbers 2069139 and FA2006040700010); North-West University, Population Health Research Institute, and the Medical Research Council of South Africa. M. E. W. was funded by the National Research Foundation, South Africa, Thuthuka grant (TTK22031652) and Poliomyelitis Research Foundation (PRF) grant (23/84). L. K. A. was funded by the National Research Foundation, South Africa Postgraduate Scholarship (grant UID number 140524) and Poliomyelitis Research Foundation grant (23/24).
